# Exploration on Bioflocculation of *Nannochloropsis oculata* Using Response Surface Methodology for Biodiesel Production

**DOI:** 10.1155/2014/202659

**Published:** 2014-02-05

**Authors:** Duraiarasan Surendhiran, Mani Vijay

**Affiliations:** Bioelectrochemical Laboratory, Department of Chemical Engineering, Faculty of Engineering and Technology, Annamalai University, Annamalai Nagar, Tamilnadu 608002, India

## Abstract

Harvesting of algal biomass in biodiesel production involves high energy input and cost incurred process. In order to overcome these problems, bioflocculation process was employed and the efficiency of this process was further improved by the addition of a cationic inducer. In this work marine *Bacillus subtilis* was used for bioflocculation of *Nannochloropsis oculata* and ZnCl_2_ as cationic inducer. This study worked under the principle of divalent cationic bridging (DCB) theory. Under temperature stress and high pH, the bacterium produced exopolysaccharide that bound with microalga *Nannochloropsis oculata* and flocculated them. A maximum efficiency of 95.43% was observed with the optimised RSM parameters—temperature 30.78°C, pH 10.8, flocculation time 6.7 h, bioflocculant size 0.38 mL, and cationic inducer concentration 0.035 mM. The present investigation focused on the cost effective harvesting of microalga on a larger scale for biodiesel production than using toxic, ecofriendly chemical flocculants.

## 1. Introduction

The world's oil production is expected to decline in next ten decades due to burgeoning population and uncontrolled urbanization that have created serious problems of energy requirement. Global warming is one of the major environmental problems occurring because of increasing CO_2_ concentration in the atmosphere due to excessive consumption of fossil fuels [1]. Thus an alternate fuel has to be generated against fossil fuel. Biodiesel, also known as fatty acid methyl esters (FAMEs), is a potential substitute for conventional diesel fuel, which is obtained by the transesterification of triglyceride with a short chain alcohol (methanol or ethanol) [2–6]. In addition, the biodiesel has more advantages over diesel fuel because of its renewability, biodegradability, and lower emission of CO_2_ [5]. Biofuels produced from crops have become a major controversy due to food versus fuel competition and animal fat cannot be considered as a continuous supply of feed stock [7], whereas algae can be grown using poor quality waters as they do not compete with food crops for arable land and water [8]. Moreover, microalgae have advantages of high growth rate and contain more amounts of lipids from 20% to 80% of dry cell weight than the conventional oil crops which produce only 5% of dry weight [9].

Considering the growing demand for energy, algae are one of the most important energy sources for future [10], because oil crops, waste cooking oils, and fats cannot meet current and future demand for biodiesel [11]. Thus, microalgae represent one of the viable and renewable sources of biodiesel feedstock that can meet global demand for transport fuels [11, 12]. Intensive cultivation for production of large quantities of microalgae biomass requires a proper harvesting technique. One of the major problems in large scale productions of microalgae is the development of efficient separation of cells from culture broth and also to maintain their viability and bioactivity prior to use in the field [13].

Because of the small size of the algal cells (3–30 *μ*m in diameter) [14], biomass harvesting in microalgae represents one of the significant cost factors in the production of biodiesel from microalgae [15–17]. Therefore, microalgae harvesting process became a challenging task and commercial production of biodiesel from microalgae is economically unfeasible. Different studies showed that the harvesting cost of algal production in open ponds accounts for more than 20–30% of the total cost of biodiesel production [18]. The potential of microalgae for biodiesel production is based on the microalgal biomass concentrate [14]. Thus, to minimize the energy consumption of harvesting microalgae, an integrated approach is needed [19]. Therefore, microalgae harvesting is one of the difficult processes thus obstructing the development of algae biodiesel.

A significant reduction in the cost of microalgal biomass production will require cost-efficient methods for harvesting microalgae [20]. Many separation methods such as centrifugation, gravity sedimentation, (ultra)filtration, and ultra sound waves have been developed for microalgae recovery. However, each has its disadvantages that affect the overall economics of the process. Centrifugation requires high energy input and initial capital cost and the process involves exposing cells to high gravitational and shear forces which damage the cell structure. Second, the processing of large culture volumes can be time-consuming. Filtration and screening require regular replacement of filters, screens, and membranes and can be very time consuming. Gravity sedimentation is a slow process and electroflotation requires replacement of worn electrodes that have high cost of electricity consumption [21].

Evaluation of several harvesting methods showed that flocculation is the most promising cost and energy efficient alternative [22, 23]. During flocculation, the dispersed microalgal cells aggregate and form flocs with higher sedimentation rate [24, 25]. In addition, it allows the handling of large volumes of cultures and cells harvested by flocculation are in better physical condition [26].

Chemical substances that are commonly used as flocculants are highly toxic to humans and nondegradable and the intermediate byproducts of degradation are also harmful to the ecosystem [16, 27, 28]. Now researches are being focused on bioflocculation agent that is advantageous over chemical flocculant due to their biodegrading nature, high efficiency, nontoxicity, and ecofriendliness [29–32]. Bioflocculants are primarily made up of polysaccharides secreted by microorganisms extracellularly. These exopolymeric substances orexopolysaccharides (EPS) are generally produced by bacteria, yeast, and fungi during their growth [33], playing a vital role in a flocculation process.

EPS produced by the bacterial culture is lesser and would be insufficient when harvesting in large scale; that is, the extracellular product from the bacterial cell is cost consuming. To overcome such a problem, we used the whole live culture as bioflocculant for harvesting *Nannochloropsis oculata* for biodiesel production. To increase the efficiency of bioflocculation, the divalent cations had been added as an inducer in the bioflocculation process to neutralize the similar net negative charges of EPS and the microalgal cell wall.

As to the best of our knowledge, there are scanty reports available on use of whole culture as bioflocculant, thus this would be one of the first reports on bioflocculation using live cells. The present investigation involved the bioflocculation process enhanced by inducer which was selected through cell viability test and optimized using Response Surface Methodology (RSM) with important physical parameters like temperature, pH, flocculation time, bioflocculant size, and cationic inducer concentration.

## 2. Materials and Methods

### 2.1. Organism and Culture Medium

*Nannochloropsis oculata*, obtained from the Central Marine and Fisheries Research Institute (CMFRI), Tuticorin, Tamilnadu (India), was grown in sterile Walne's medium. The filtered sterilized sea water was enriched with required quantity of Walne's medium composition containing (g L^−1^): NaNO_3_, 100; NaH_2_PO_4_ · 2H_2_O, 20.0; Na_2_EDTA, 4.0; H_3_BO_3_, 33.6; MnCl_2_ · 4H_2_O, 0.36; FeCl_3_ · 6H_2_O, 13.0; vitamin B_12_, 0.001 and vitamin B_1_, 0.02. The trace metal solution contained (g L^−1^): ZnSO_4_ · 7H_2_O, 4.4; CoCl_2_ · 6H_2_O, 2.0; (NH_4_)_6_Mo_7_O_24_ · H_2_O, 0.9; and CuSO_4_ · 5H_2_O, 2.0. The medium was adjusted to pH 8 and autoclaved at 121°C for 20 min. The filter sterilized vitamins were added after cooling. The contents were later introduced into a 250 mL Erlenmeyer flask and finally transferred to 25 L photobioreactor (PBR). Mixing was provided by sparging air from the bottom of the PBR; lighting was supplied by cool-white fluorescent tubes with an intensity of 5000 lux. End of the log phase culture was used for the coagulation experiments.

### 2.2. Culture for Bioflocculation

The marine bacterial culture *Bacillus subtilis* (MTCC 10619) was used as the bioflocculant, obtained from the Department of Marine Biology, Parangipettai, Annamalai University, India. The bacterial culture was cultivated for growth and bioflocculant production using nutrient broth supplemented with 3% NaCl subcultured periodically and stored as stocks on nutrient agar slants at 4°C.

### 2.3. Evaluation of Bioflocculation Experiment: One-Factor-at-a-Time Design

Flocculation experiments were carried out in stationary growth phase of microalgae. A quantity of 50 mL of *Nannochloropsis oculata* was used for optimization study. The effects of bioflocculation parameters, namely, temperature, pH, time, bioflocculant concentration, and cationic inducer size, were individually experimented by analyzing flocculation efficiency. For the effect of pH, the culture was divided in a series of test tubes, and the pH was adjusted to fixed values by the addition of 1 M HCl or 1 M NaOH, ranging from approximately 6.0 to 10. Likewise, for effect of temperature the test tubes were incubated at desired temperatures. After the parameter setup, each tube was kept in orbital shaker (Model-Technico, Honeywell Ltd, India) and stirring speed was maintained at 250 rpm. The initial microalgal biomass concentration in the tubes was estimated from the optical density of 750 nm (OD_750_) in UV-VIS Spectrophotometer (Model-SL 159, ELICO Ltd, India). At the end of the bioflocculation time, the optical density of the supernatant was measured at half the height of the clarified culture. Culture broth containing no bioflocculant was used as control. Bioflocculation efficiency was calculated by the following [34, 35]:

(1) $$\begin{array}{c} \;\text{Flocculation}\;\text{Efficiency}\left(\%\right)=\left(1-\frac{A}{B}\right)\times 100, \end{array}$$

where *A* = OD_750_ value of sample and *B* = OD_750_ value of control.

### 2.4. Response Surface Methodology: CCD

A central composite design (CCD) of the experiments was formulated to investigate five flocculation parameters. Each 50 mL culture of *Nannochloropsis oculata *was added into test tubes and the parameters were set according to the orthogonal values of central composite design (CCD) (Table 1). RSM is known to evaluate the interaction between the significant factors of an experiment and optimize them [23]. Five-level factor experiment setup was designed using Design Expert Software version 8.0.7.1, Stat-Ease, Minneapolis, USA, and the quality of analysis model was based on an analysis of variance (ANOVA). The response variable (*Y*), representing the bioflocculation activity, was fitted using a second-order polynomial equation given as

(2) Y=β0+β1X1+β2X2+β3X3+β4X4+β5X5+β12X1X2+β13X1X3+β14X1X4+β15X1X5+β23X2X3+β24X2X4+β25X2X5+β34X3X4+β35X3X5+β45X4X5+β11X12+β22X22+β33X32+β44X42+β55X52,

where *Y* is the predicted response, *β*_0_ was the constant, *X*_1_–*X*_5_ were the input variables, *β*_1_–*β*_5_ were the linear coefficients, *β*_12_–*β*_45_ were the second order interactive coefficients, and *β*_11_–*β*_45_ were the quadratic coefficients.

The actual value of coded levels of different parameters which are temperature (*X*_1_), pH (*X*_2_), flocculation time (*X*_3_), bioflocculant size (*X*_4_), and cationic inducer concentration (*X*_5_) is presented in Table 1 and its influence on harvesting of microalgae by flocculation, represented as *Y*, the response variable, has been investigated. The actual values of coded level “0” were fixed based on one-factor-at-a-time method.

## 3. Results and Discussion

### 3.1. Variables Influencing the Bioflocculation Process

#### 3.1.1. Effect of Temperature on Bioflocculation

The flocculation efficiency reached its maximum as the temperature was increased till 30°C, after which the flocculation efficiency decreased (Figure 2). Effective process occurred at a temperature of 30°C, as the cells of marine bacterium, *B. subtilis* (MTCC 10619), were able to produce more bioflocculant, that is, exopolysaccharide (EPS) at high pH stress condition. A rapid decrease in efficiency was observed, when the temperature was raised beyond 30°C, which was due to the susceptibility of microalgae cells as well as molecular mobility at higher temperature. Thus collision occurred between bioflocculant and microalgal cells, which lead to cell distortion [36]. Moreover, as the microalgae and the bioflocculant producing bacteria are from marine sources, supplementation of additional medium components/nutrients may not be necessary.

#### 3.1.2. Effect of pH on Bioflocculation

pH is one of the most important factors for harvesting microalgae; hence the influence of pH on bioflocculation efficiency was tested with a pH range from 6 to 10. From the statistical experimental results, the effect of pH on flocculation efficiency was highly significant (*P* < 0.01) and the flocculation efficiency was found to be higher with increase in pH, that is, 10. This result is in agreement with previous studies [20]. As pH increases, the negative charge of microalgal cells increases. This phenomenon could be a major cause for flocculation by higher pH. This is due to difference in protonation conformational changes and structural alterations in flocs.

#### 3.1.3. Effect of Bioflocculant Size on Bioflocculation

**Figure 1:**
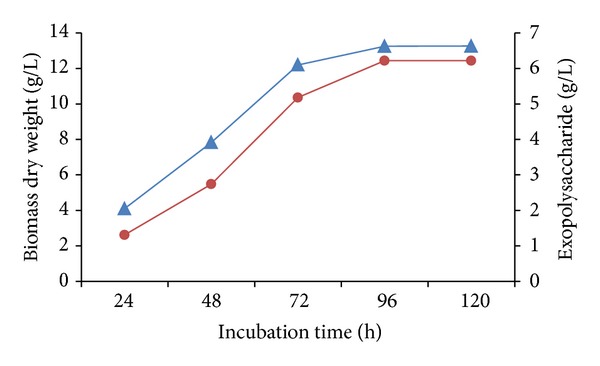
Time course of batch culture of *Bacillus subtilis* MTCC10619. Blue triangles: cell dry weight (g/L) and red bullets: bioflocculant production (g/L).

As the time prolonged, deterioration was observed in bioflocculation efficiency. Bioflocculants (EPS) are generally found to be produced during late exponential phase or stationary phase of the bacterial growth (Figure 1) [22], after which the concentration or the production of the polymer remains constant in the medium. Hence as the time increased beyond the production time, flocculation decreased. Lower efficiency could also be experienced when cells produce deflocculating enzymes, along with bioflocculants, beyond stationary phase [22].

Significant effect was observed with bioflocculant size on flocculation of *N. oculata*. The more was the bioflocculant, the lesser was the interaction between them and the lower was the efficiency. The flocculation mechanism between microalgal cells and bacterial cells happened as a series of interbridging between cells, neutralization of charges, and precipitation enmeshment [23]. Larger amount of bioflocculant might be detrimental, due to its adsorption to the cells, reducing their surface potential and destabilizing the microalgal cells. Similarly, harvesting microalgal cells using *γ*-poly glutamic acid, on overdosing with 30 mg/L, caused lower flocculation due to charge neutralization and destabilization [23].

#### 3.1.4. Role of Cationic Inducer

**Figure 2:**
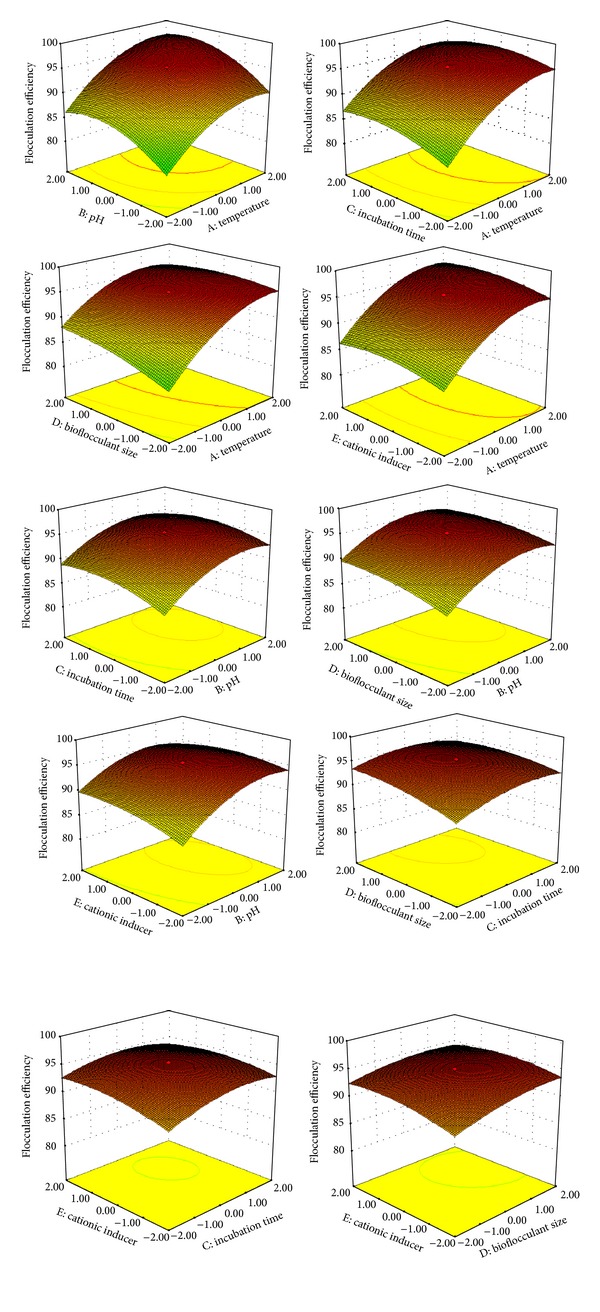
3D Response surface and contour plots representing various interactive effects of variables on bioflocculation.

For this study, ZnCl_2_ was used as cationic inducer, as confirmed by cell viability test carried out in our previous study [21]. Trace amount of ZnCl_2_ added as cationic inducer significantly affected bioflocculation. Addition of divalent cationic salts in the medium enhanced the flocculation at high pH by interlinking the cells, forming dense flocs [22] because the exopolymer produced by *B. subtilis* was negatively charged similar to the microalgal cell wall. This mechanism is known as “divalent cation bridging theory (DCB)” by Sobeck and Higgins in 2002 [39], used in waste water treatment, which explains the improvement in floc properties. Zn^2+^ salt aides the process as a linker, which neutralizes the residual negative charge of functional groups thus enhancing the bioflocculation process [30]. Higher concentration of the inducer led to the destruction of the compact conformation of the cells, and flocculation efficiency became lesser (Figure 2).

#### 3.1.5. Central Composite Design

Bioflocculation of *N. oculata* was carried out with marine *B. subtilis* producing EPS. Optimization of the five independent variables was performed using central composite design (CCD) with 50 runs and 7 central points. The predicted and experimental responses from each experiment were tabulated (Table 2). A positive sign denoted that the effect of the variables on flocculation was greater at a higher concentration whereas a negative symbol represented that influence of variable on flocculation is greater at a lower concentration.

Multiple linear regression analysis was carried out using a second-order polynomial equation that was fitted to the above data as

(3) Ybiofloc=96.5512+3.65938X1+1.33237X2+1.15548X3+1.18964X4+1.07491X5−2.87281X12−1.05555X22−1.1183X32−1.22437X42−1.20492X52−0.43968X1X2−0.04093X1X3−0.16281X1X4−0.58843X1X5−0.20093X2X3−0.37281X2X4+0.37406X2X5−0.45406X3X4−0.12468X3X5−0.271563X4X5,

where *Y*_biofloc_ is the response variable, *X*_1_ to *X*_2_ are the linear effects of the independent variables such as temperature, pH, flocculation time, bioflocculant size, and cationic inducer size, respectively, *X*_1_, *X*_2_ to *X*_4_, *X*_5_ are the interactive terms of the variables, and *X*_1_^2^ to *X*_5_^2^ are squared effects of the variables.

The variation of different parameters which are temperature (*X*_1_), pH (*X*_2_), flocculation time (*X*_3_), bioflocculant size (*X*_4_), and cationic inducer concentration (*X*_5_) is presented in Table 1 and its influence on harvesting of microalgae, which represents response variable (*Y*), has been investigated.

The goodness of fit of regression equation developed could be measured by determination coefficient. The *R* ^2^ value of 0.8648 and adjusted *R* ^2^ of 0.7715 showed that the model could be significant predicting the response and explaining 95% of the variability in the data.  Table 4 revealed the statistical significance of each coefficient. Smaller probability (*P*) values, that is, lesser than 0.05 (*P* < 0.05) and larger magnitude of “*t*” values indicate the significance of the model. The coefficients of this response, namely, *X*_1_, *X*_2_, *X*_3_, *X*_4_, *X*_5_, *X*_1_^2^, *X*_2_^2^, *X*_3_^2^, *X*_4_^2^, *X*_5_^2^, *X*_1_ *X*_2_, *X*_1_ *X*_5_, *X*_2_ *X*_4_, *X*_2_ *X*_5_, and *X*_3_ *X*_4_ were found to be most significant of this model (*P* < 0.05). ANOVA table illustrated (Table 3) the calculated *F* value (9.27) and a low *P* value (*P* = 0.0001) demonstrated that the quadratic model was highly significant.

Three dimensional response surface plots and contour plots for the bioflocculation efficiency were shown in Figure 2. The shapes of the contour plots indicate the significance of the interaction between the variables. An elliptical plot illustrates greater significance of interaction whereas a circular contour plot indicates that the interaction is negligible [40–42]. Mutual interactions and optimization of the tested variables could be conveniently studied through 3D surface and contour plots. From the graphical representation the effects of interactions were studied.

By analyzing the response surface plots and contour representation, the optimal values of the tested variables for the highest bioflocculation efficiency were temperature 30.78°C, pH 10.8, flocculation time 6.7 h, bioflocculant size 0.38 mL, and cationic inducer concentration 0.036 mM. This model also exhibited that the interaction between variables was also highly significant. The coefficients *X*_1_ *X*_2_, *X*_1_ *X*_5_, *X*_2_ *X*_4_, *X*_2_ *X*_5_, and *X*_3_ *X*_4_ (*P* < 0.05) were found to be highly significant model terms. The significant interaction could be clearly observed from the elliptical contour plots. The validation of the model was done by carrying out in triplicates under optimized conditions. The mean value obtained was 95.56%, which was in good agreement with the predicted response.

Our current findings indicated that the bioflocculation process under the optimized conditions gave the maximum efficiency as 95.43% whereas various other flocculation procedures produced lower results (Table 4). Thus, our study concluded that bioflocculation using live whole bacterial cells producing exopolysaccharide bioflocculant is highly efficient to harvest microalgal cells for biodiesel production.

## 4. Conclusions

The present study dealt with the harvesting of marine microalga, *Nannochloropsis oculata, *using a natural flocculant marine bacterium *Bacillus subtilis. *The bacteria, major producer of exopolysaccharide, influenced flocculation to a greater extent. Microbial flocculant required a very low concentration of 0.035 mM chemical flocculating agent, ZnCl_2_ for the process to be enhanced and efficient. Using RSM the variables were statistically optimised which resulted in 95.43% flocculation efficiency with 0.38 mL of bioflocculant. Through the study the microbial source for flocculation was explored to be essentially a better replacement of synthetic flocculant, thereby providing a potential source for ecofriendly and cost effective large scale harvesting of microalga for biodiesel production.

## Figures and Tables

**Table 1:** Coded values based on the factor at a time experiment for the 5 variables employed in the study.

Code	Variables	−2	−1	0	+1	+2
*X*_1_	Temperature (°C)	20	25	30	35	40
*X*_2_	pH	6	7	8	9	10
*X*_3_	Flocculation time (hr)	2	4	6	8	10
*X*_4_	Bioflocculant size (mL)	0.1	0.2	0.3	0.4	0.5
*X*_5_	Cationic inducer concentration (mM)	0.01	0.02	0.03	0.04	0.05

**Table 2:** Central composite design matrix of orthogonal values with observed responses on bioflocculation efficiency.

Run	*X*_1_	*X*_2_	*X*_3_	*X*_4_	*X*_5_	Bioflocculation efficiency (%)	
						Experimental value	Predicted value
1	35	9	8	0.2	0.02	93.43	94.36
2	25	7	8	0.4	0.02	85.39	81.56
3	35	7	8	0.2	0.04	92.79	89.16
4	35	9	4	0.4	0.04	91.33	94.46
5	35	7	4	0.2	0.04	88.78	87.55
6	25	7	8	0.4	0.04	80.00	82.51
7	35	7	8	0.4	0.02	83.81	86.53
8	35	7	4	0.2	0.02	79.33	82.25
9	30	8	6	0.5	0.03	94.02	92.80
10	30	8	6	0.3	0.03	95.43	95.00
11	30	8	2	0.3	0.03	83.11	83.69
12	35	9	4	0.4	0.02	92.83	93.95
13	25	7	8	0.2	0.02	76.47	76.10
14	35	7	8	0.2	0.02	81.45	85.91
15	25	9	4	0.4	0.02	85.44	84.18
16	40	8	6	0.3	0.03	92.08	88.55
17	30	8	6	0.1	0.03	90.41	86.35
18	25	7	4	0.4	0.04	83.12	80.84
19	30	8	10	0.3	0.03	94.10	88.23
20	25	7	4	0.2	0.02	74.11	69.71
21	35	9	8	0.4	0.02	94.34	93.43
22	35	9	4	0.2	0.04	94.22	94.50
23	35	9	4	0.2	0.02	92.66	92.22
24	25	9	4	0.4	0.04	85.21	84.16
25	30	10	6	0.3	0.03	92.42	85.67
26	25	9	4	0.2	0.04	77.23	79.36
27	25	9	8	0.4	0.04	83.90	84.32
28	25	9	8	0.4	0.02	79.54	86.39
29	25	9	8	0.2	0.02	80.12	82.49
30	25	9	8	0.2	0.04	84.98	82.20
31	25	7	4	0.4	0.02	74.48	77.84
32	35	7	4	0.4	0.04	88.79	89.07
33	35	7	4	0.4	0.02	86.90	85.55
34	25	7	4	0.2	0.04	73.02	74.48
35	35	9	8	0.2	0.04	87.65	94.59
36	25	7	8	0.2	0.04	73.75	78.82
37	30	6	6	0.3	0.03	70.19	71.66
38	20	8	6	0.3	0.03	66.39	64.64
39	30	8	6	0.3	0.05	92.49	90.62
40	25	9	4	0.2	0.02	72.99	77.61
41	35	7	8	0.4	0.04	88.53	88.01
42	30	8	6	0.3	0.01	90.20	86.78
43	35	9	8	0.4	0.04	89.52	91.89

**Table 3:** ANOVA table for response surface function on bioflocculation efficiency.

Source	DF	SS	MS	*F*	*P*
Regression	20	2725.56	136.28	9.27	<0.0001
Linear	5	1617.27	323.45	0.31	0.9740
Square	10	127.39	12.74	13.35	<0.0001
Interaction	5	980.90	196.18	0.75	0.7045
Residual Error	29	426.21	14.70		

Total	49	3151.77			

DF: degree of freedom; SS: sum of squares; MS: mean square; *F*: Fischer's value; *P*: probability value.

**Table 4:** Comparison of different harvesting methods and their efficiencies.

Method	Microalgae	Harvesting efficiency (%)	References
Bioflocculation with whole cell	*Nannochloropsis oculata *	>95	Current study
Flocculation with polyelectrolytes	*Chaetoceros calcitrans *	>90	[13]
Flocculation with *γ*-poly glutamic acid	*Chlorella vulgaris *and* Nannochloropsis oculata *	>90	[23]
Flocculation with AlCl_3_	*Chlorella minutissima *	>90	[35]
Flocculation with cationic polymer	*Chlorococcum *sp*. *	>89	[36]
Flocculation with chitosan	*Thalassiosira pseudonana *	90	[37]
Centrifugation	*Phaeodactylum tricornutum *	94	[37]
Increasing pH	*Dunaliella tertiolecta *	90	[38]
